# 4′-(4-Methoxy­phen­yl)-1,1′,1′′-trimethyl­dispiro­[indoline-3,2′-pyrrolidine-3′,3′′-pyrrolidine]-2,2′′,5′′-trione

**DOI:** 10.1107/S1600536809021114

**Published:** 2009-06-20

**Authors:** S. Nirmala, K. Karthikeyan, E. Theboral Sugi Kamala, L. Sudha, P. T. Perumal

**Affiliations:** a Department of Physics, Easwari Engineering College, Ramapuram, Chennai 600 089, India; b Organic Chemistry Division, Central Leather Research Institute, Adyar, Chennai 600 020, India; c Department of Physics, SRM University, Ramapuram Campus, Chennai 600 089, India

## Abstract

In the title compound, C_24_H_25_N_3_O_4_, the pyrrolidine ring adopts an envelope conformation while the pyrrolidine-2′′,5′′-dione ring adopts a twist conformation. The indoline unit is planar [maximum deviation of −0.050 (9) Å] and forms a dihedral angle of 40.36 (4)° with the methoxy­phenyl ring. Intra­molecular C—H⋯O hydrogen bonds are observed. In the crystal, mol­ecules are linked into a two-dimensional network parallel to the *ab* plane by inter­molecular C—H⋯O hydrogen bonds and C—H⋯π inter­actions.

## Related literature

For the biological activity of spiro-pyrrolidine-containing compounds, see: Araki *et al.* (2002 ▶); Gore *et al.* (1991 ▶); James *et al.* (1991 ▶); Kobayashi *et al.* (1991 ▶); Tietze *et al.* (1988 ▶). For the biological activity of indole derivatives, see: Harris & Uhle (1960 ▶); Ho *et al.* (1986 ▶); Stevenson *et al.* (2000 ▶). For a related structure, see: Govind *et al.* (2003 ▶). For ring-puckering parameters, see: Cremer & Pople (1975 ▶).
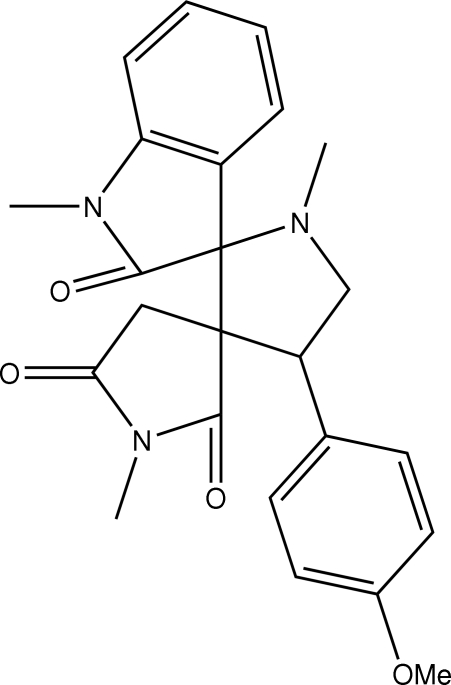


## Experimental

### 

#### Crystal data

C_24_H_25_N_3_O_4_
*M* *_r_* = 419.47
Orthorhombic, 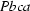

*a* = 11.2074 (3) Å
*b* = 11.2406 (3) Å
*c* = 33.6082 (9) Å
*V* = 4233.9 (2) Å^3^
*Z* = 8
Mo *K*α radiation
μ = 0.09 mm^−1^
*T* = 293 K
0.25 × 0.17 × 0.15 mm

#### Data collection

Bruker Kappa APEXII area-detector diffractometer
Absorption correction: multi-scan (Blessing, 1995 ▶) *T*_min_ = 0.978, *T*_max_ = 0.987
17736 measured reflections
5023 independent reflections
3269 reflections with *I* > 2σ(*I*)
*R*_int_ = 0.028

#### Refinement

*R*[*F* ^2^ > 2σ(*F* ^2^)] = 0.049
*wR*(*F* ^2^) = 0.152
*S* = 1.07
5023 reflections
284 parameters
H-atom parameters constrained
Δρ_max_ = 0.24 e Å^−3^
Δρ_min_ = −0.18 e Å^−3^

### 

Data collection: *APEX2* (Bruker, 2004 ▶); cell refinement: *SAINT* (Bruker, 2004 ▶); data reduction: *SAINT*; program(s) used to solve structure: *SHELXS97* (Sheldrick, 2008 ▶); program(s) used to refine structure: *SHELXL97* (Sheldrick, 2008 ▶); molecular graphics: *ORTEP-3* (Farrugia, 1997 ▶); software used to prepare material for publication: *PLATON* (Spek, 2009 ▶).

## Supplementary Material

Crystal structure: contains datablocks I, global. DOI: 10.1107/S1600536809021114/ci2815sup1.cif

Structure factors: contains datablocks I. DOI: 10.1107/S1600536809021114/ci2815Isup2.hkl

Additional supplementary materials:  crystallographic information; 3D view; checkCIF report

## Figures and Tables

**Table 1:** Hydrogen-bond geometry (Å, °)

*D*—H⋯*A*	*D*—H	H⋯*A*	*D*⋯*A*	*D*—H⋯*A*
C10—H10⋯O2	0.93	2.52	3.122 (2)	123
C12—H12*A*⋯O1	0.97	2.58	3.222 (2)	124
C20—H20⋯O1	0.93	2.56	3.400 (2)	150
C12—H12*B*⋯O1^i^	0.97	2.56	3.264 (2)	129
C16—H16⋯O4^ii^	0.93	2.50	3.394 (2)	161
C19—H19⋯O2^iii^	0.93	2.54	3.440 (2)	162
C21—H21*C*⋯*Cg*1^iv^	0.96	2.93	3.592 (2)	127

Symmetry codes: (i) 
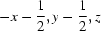
; (ii) 
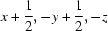
; (iii) 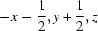; (iv) 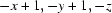. *Cg*1 is the centroid of the C15–C20 ring.

**Fig. 1.**
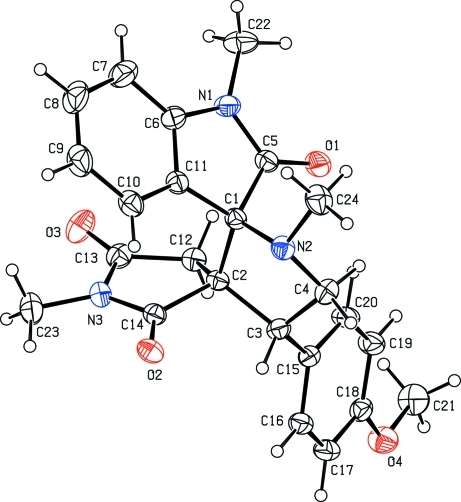
The molecular structure of the title compound with 30% probability displacement ellipsoids.

**Fig. 2.**
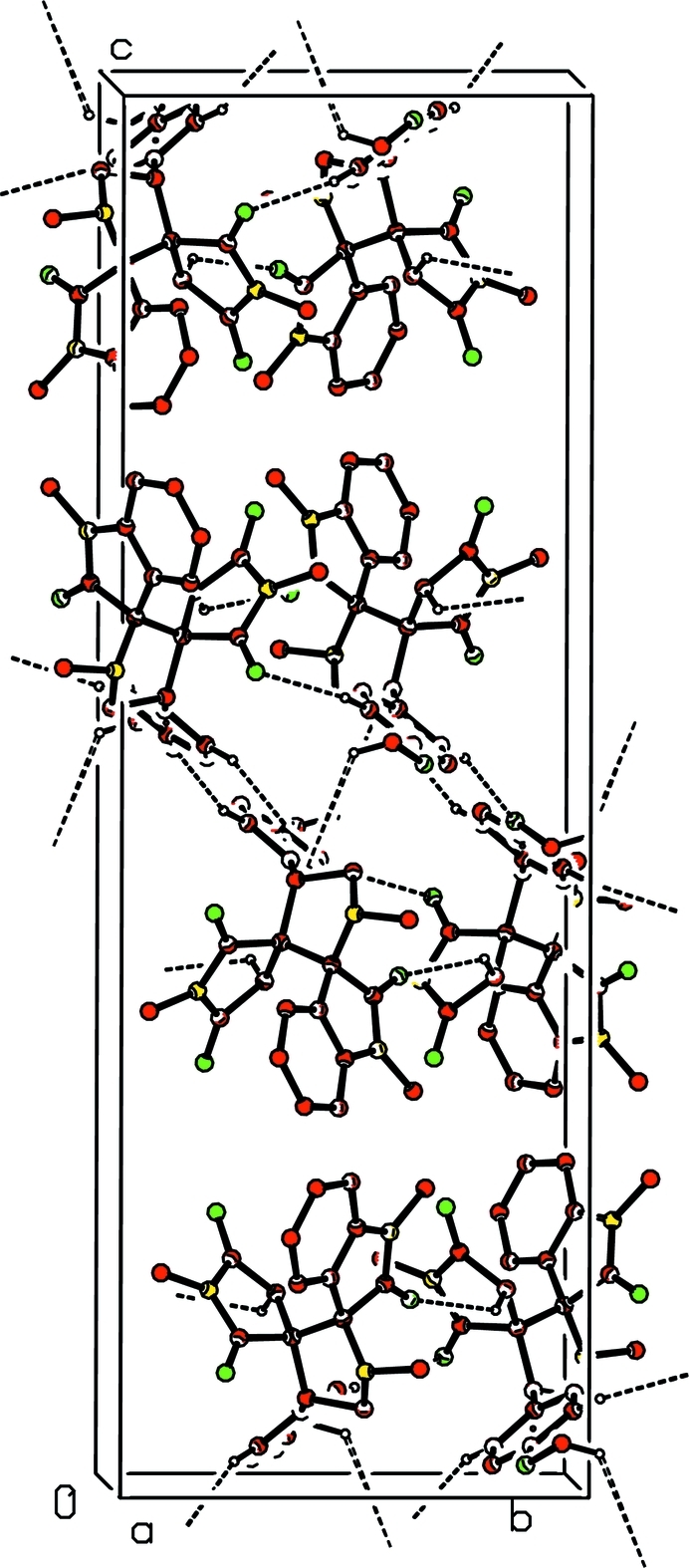
The packing of the molecules viewed down the a axis. Dashed lines indicate hydrogen bonds. H atoms not involed in hydrogen bonds have been omitted.

## supplementary crystallographic information

### Comment

Spiro compounds are a particular class of naturally occurring substances characterized by highly pronounced biological properties (Kobayashi *et al.*, 1991; James *et al.*, 1991). The spiro-pyrrolidine ring system is also found in phermones, antibiotics (Gore *et al.*, 1991) and antitumour agents (Tietze *et al.*, 1988; Araki *et al.*, 2002).

Indole compounds can be used as bioactive drugs (Stevenson *et al.*, 2000). Indole derivatives exhibit antiallergic, central nervous system depressant and muscle relaxant properties (Harris & Uhle, 1960; Ho *et al.*, 1986). In view of this biological importance, the crystal structure of the title compound was determined and the results are presented here.

An *ORTEP* (Farrugia,1997) plot of the molecule is shown in Fig.1. The indole unit is planar, with a maximum deviation of -0.050 (9) Å for atom C1. The dihedral angle between the indole unit and the methoxyphenyl ring is 40.36 (4)°. The sum of angles at atoms N1 (360.0°) and N3 (359.9°) is in accordance with *sp*^2^ hybridization, whereas the sum of angles at N2 (335.5°) is in accordance with *sp*^3^ hybridization. The N1—C5 and C5—O1 bond lengths show electron delocalization over atoms N1, C5 and O1. In the oxindole ring system, the variation in endocyclic angles are due to the fusion of the five- and six-membered rings (Govind *et al.*, 2003). The methoxy group is almost coplanar with the C15-C20 benzene ring [C21—O4—C18—C17 = 174.7 (2)°]. The N2/C1-C4 pyrrolidine ring adopts an envelope conformation with puckering parameters q_2_ and φ of 0.404 (2) Å and -38.8 (2)° respectively (Cremer & Pople, 1975). Atom N2 deviates by 0.590 Å from the least-squares plane through the remaining four atoms. The N3/C2/C12-C14 pyrrolidine ring adopts a twist conformation, with puckering parameters q_2_ and φ of 0.233 (2) Å and -13.2 (4)° respectively (Cremer & Pople, 1975).

The molecular structure is stabilized by intramolecular C— H···O hydrogen bonds and the crystal packing is determined by intermolecular C—H···O hydrogen bonds and C—H···π interactions involving C15—C20 benzene rings (Table 1). In addition the packing is stabilized by van der Waals forces.

### Experimental

A mixture of sarcosine (1 mmol), 1-methylisatin (1 mmol) and 3-(4-methoxybenzylidine)-1-methyl-pyrrolidine-2,5-dione (1 mmol) was refluxed in methanol. Completion of the reaction was evidenced by TLC analysis. The solvent was then removed *in vacuo* and the crude product subjected to column chromatography (100–200 mesh) using petroleum ether–ethyl acetate as eluent. Single crystals were obtained by crystallization from petroleum ether and ethyl acetate mixture.

### Refinement

H atoms were placed in idealized positions and allowed to ride on their parent atoms, with C—H = 0.93, 0.98, 0.97 and 0.96 Å for aromatic, methine, methylene and methyl H respectively, and *U*_iso_(H) = 1.5U_eq_(C) for methyl H and *U*_iso_(H) = 1.2U_eq_(C) for all other H atoms.

### Crystal data

**Table:**

C_24_H_25_N_3_O_4_	*F*(000) = 1776
*M**_r_* = 419.47	*D*_x_ = 1.316 Mg m^−^^3^
Orthorhombic, *P**b**c**a*	Mo *K*α radiation, λ = 0.71073 Å
Hall symbol: -P 2ac 2ab	Cell parameters from 5049 reflections
*a* = 11.2074 (3) Å	θ = 2.4–25.7°
*b* = 11.2406 (3) Å	µ = 0.09 mm^−^^1^
*c* = 33.6082 (9) Å	*T* = 293 K
*V* = 4233.9 (2) Å^3^	Prism, colourless
*Z* = 8	0.25 × 0.17 × 0.15 mm

### Data collection

**Table:**

Bruker Kappa APEXII area-detector diffractometer	5023 independent reflections
Radiation source: fine-focus sealed tube	3269 reflections with *I* > 2σ(*I*)
graphite	*R*_int_ = 0.028
ω and φ scans	θ_max_ = 28.4°, θ_min_ = 1.2°
Absorption correction: multi-scan (Blessing, 1995)	*h* = −14→11
*T*_min_ = 0.978, *T*_max_ = 0.987	*k* = −15→11
17736 measured reflections	*l* = −43→43

### Refinement

**Table:**

Refinement on *F*^2^	Primary atom site location: structure-invariant direct methods
Least-squares matrix: full	Secondary atom site location: difference Fourier map
*R*[*F*^2^ > 2σ(*F*^2^)] = 0.049	Hydrogen site location: inferred from neighbouring sites
*wR*(*F*^2^) = 0.152	H-atom parameters constrained
*S* = 1.07	*w* = 1/[σ^2^(*F*_o_^2^) + (0.0744*P*)^2^ + 0.5831*P*] where *P* = (*F*_o_^2^ + 2*F*_c_^2^)/3
5023 reflections	(Δ/σ)_max_ = 0.001
284 parameters	Δρ_max_ = 0.24 e Å^−^^3^
0 restraints	Δρ_min_ = −0.18 e Å^−^^3^

### Special details

**Table:**

Geometry. All e.s.d.'s (except the e.s.d. in the dihedral angle between two l.s. planes) are estimated using the full covariance matrix. The cell e.s.d.'s are taken into account individually in the estimation of e.s.d.'s in distances, angles and torsion angles; correlations between e.s.d.'s in cell parameters are only used when they are defined by crystal symmetry. An approximate (isotropic) treatment of cell e.s.d.'s is used for estimating e.s.d.'s involving l.s. planes.
Refinement. Refinement of *F*^2^ against ALL reflections. The weighted *R*-factor *wR* and goodness of fit *S* are based on *F*^2^, conventional *R*-factors *R* are based on *F*, with *F* set to zero for negative *F*^2^. The threshold expression of *F*^2^ > σ(*F*^2^) is used only for calculating *R*-factors(gt) *etc*. and is not relevant to the choice of reflections for refinement. *R*-factors based on *F*^2^ are statistically about twice as large as those based on *F*, and *R*- factors based on ALL data will be even larger.

### Fractional atomic coordinates and isotropic or equivalent isotropic displacement parameters (Å^2^)

**Table:**

	*x*	*y*	*z*	*U*_iso_*/*U*_eq_	
C1	0.01846 (15)	0.47299 (15)	0.12817 (5)	0.0327 (4)	
C2	−0.06510 (14)	0.36884 (14)	0.11357 (5)	0.0316 (4)	
C3	−0.09503 (15)	0.40199 (16)	0.06949 (5)	0.0365 (4)	
H3	−0.0535	0.3440	0.0527	0.044*	
C4	−0.03321 (17)	0.52114 (18)	0.06291 (5)	0.0433 (5)	
H4A	−0.0046	0.5282	0.0358	0.052*	
H4B	−0.0868	0.5868	0.0685	0.052*	
C5	−0.05564 (15)	0.56643 (16)	0.15170 (5)	0.0357 (4)	
C6	0.08728 (16)	0.49862 (17)	0.19406 (6)	0.0408 (4)	
C7	0.1549 (2)	0.4819 (2)	0.22787 (7)	0.0582 (6)	
H7	0.1375	0.5215	0.2515	0.070*	
C8	0.2500 (2)	0.4037 (2)	0.22515 (7)	0.0665 (7)	
H8	0.2959	0.3885	0.2476	0.080*	
C9	0.2780 (2)	0.3480 (2)	0.18998 (7)	0.0608 (6)	
H9	0.3434	0.2972	0.1888	0.073*	
C10	0.20970 (17)	0.36673 (17)	0.15631 (6)	0.0456 (5)	
H10	0.2295	0.3301	0.1324	0.055*	
C11	0.11189 (15)	0.44049 (15)	0.15873 (5)	0.0353 (4)	
C12	−0.16608 (15)	0.33962 (16)	0.14310 (5)	0.0371 (4)	
H12A	−0.1860	0.4087	0.1591	0.045*	
H12B	−0.2371	0.3133	0.1291	0.045*	
C13	−0.11700 (16)	0.24208 (18)	0.16851 (6)	0.0425 (4)	
C14	0.00594 (15)	0.25280 (16)	0.11418 (5)	0.0350 (4)	
C15	−0.22493 (16)	0.39732 (16)	0.05782 (5)	0.0364 (4)	
C16	−0.26396 (17)	0.30838 (16)	0.03233 (5)	0.0402 (4)	
H16	−0.2092	0.2538	0.0224	0.048*	
C17	−0.38205 (17)	0.29936 (17)	0.02148 (6)	0.0443 (5)	
H17	−0.4061	0.2395	0.0041	0.053*	
C18	−0.46497 (16)	0.37864 (17)	0.03623 (5)	0.0385 (4)	
C19	−0.42807 (17)	0.46898 (18)	0.06074 (6)	0.0455 (5)	
H19	−0.4829	0.5242	0.0702	0.055*	
C20	−0.30887 (17)	0.47782 (18)	0.07131 (6)	0.0467 (5)	
H20	−0.2848	0.5395	0.0879	0.056*	
C21	−0.67018 (18)	0.4326 (2)	0.04175 (7)	0.0595 (6)	
H21A	−0.6672	0.4262	0.0702	0.089*	
H21B	−0.7469	0.4068	0.0324	0.089*	
H21C	−0.6575	0.5139	0.0341	0.089*	
C22	−0.0594 (2)	0.6521 (2)	0.21968 (7)	0.0586 (6)	
H22A	−0.1135	0.7077	0.2077	0.088*	
H22B	0.0048	0.6945	0.2322	0.088*	
H22C	−0.1011	0.6058	0.2393	0.088*	
C23	0.0372 (2)	0.0838 (2)	0.16109 (7)	0.0633 (6)	
H23A	0.0928	0.1050	0.1816	0.095*	
H23B	0.0795	0.0491	0.1391	0.095*	
H23C	−0.0193	0.0274	0.1713	0.095*	
C24	0.12923 (19)	0.63178 (18)	0.09418 (7)	0.0540 (5)	
H24A	0.0752	0.6924	0.1031	0.081*	
H24B	0.1609	0.6532	0.0686	0.081*	
H24C	0.1934	0.6240	0.1129	0.081*	
N1	−0.01162 (14)	0.57366 (14)	0.18924 (4)	0.0424 (4)	
N2	0.06588 (13)	0.51895 (13)	0.09103 (4)	0.0381 (4)	
N3	−0.02527 (14)	0.18934 (13)	0.14773 (5)	0.0416 (4)	
O1	−0.13847 (12)	0.62471 (12)	0.13906 (4)	0.0506 (4)	
O2	0.07934 (11)	0.21993 (11)	0.09054 (4)	0.0455 (3)	
O3	−0.14963 (13)	0.21122 (15)	0.20112 (4)	0.0626 (4)	
O4	−0.58015 (12)	0.36018 (13)	0.02477 (4)	0.0529 (4)	

### Atomic displacement parameters (Å^2^)

**Table:**

	*U*^11^	*U*^22^	*U*^33^	*U*^12^	*U*^13^	*U*^23^
C1	0.0316 (9)	0.0305 (9)	0.0360 (9)	0.0035 (7)	−0.0024 (7)	−0.0031 (7)
C2	0.0300 (8)	0.0310 (8)	0.0337 (9)	0.0008 (7)	0.0001 (7)	−0.0048 (7)
C3	0.0377 (9)	0.0411 (10)	0.0308 (9)	0.0030 (8)	−0.0017 (7)	−0.0047 (8)
C4	0.0462 (11)	0.0472 (11)	0.0365 (10)	−0.0004 (9)	−0.0011 (8)	0.0048 (9)
C5	0.0345 (9)	0.0329 (9)	0.0397 (10)	0.0020 (8)	−0.0012 (8)	−0.0045 (8)
C6	0.0390 (10)	0.0426 (10)	0.0409 (11)	−0.0025 (8)	−0.0057 (8)	−0.0017 (8)
C7	0.0603 (13)	0.0691 (15)	0.0453 (12)	−0.0024 (12)	−0.0145 (10)	−0.0015 (11)
C8	0.0634 (14)	0.0700 (16)	0.0662 (16)	0.0002 (13)	−0.0310 (13)	0.0131 (13)
C9	0.0487 (12)	0.0501 (12)	0.0835 (17)	0.0085 (10)	−0.0224 (12)	0.0048 (12)
C10	0.0367 (10)	0.0390 (10)	0.0611 (12)	0.0032 (8)	−0.0081 (9)	−0.0028 (9)
C11	0.0318 (9)	0.0318 (9)	0.0423 (10)	−0.0023 (7)	−0.0050 (8)	−0.0006 (8)
C12	0.0317 (9)	0.0398 (10)	0.0400 (10)	−0.0002 (8)	0.0002 (8)	−0.0027 (8)
C13	0.0392 (10)	0.0468 (11)	0.0416 (11)	−0.0076 (9)	−0.0038 (9)	0.0011 (9)
C14	0.0350 (9)	0.0315 (9)	0.0385 (10)	−0.0002 (7)	−0.0022 (8)	−0.0044 (8)
C15	0.0407 (10)	0.0363 (9)	0.0323 (9)	0.0044 (8)	−0.0059 (8)	−0.0029 (8)
C16	0.0436 (10)	0.0376 (10)	0.0394 (10)	0.0054 (8)	−0.0029 (8)	−0.0079 (8)
C17	0.0499 (11)	0.0392 (10)	0.0438 (11)	−0.0027 (9)	−0.0081 (9)	−0.0104 (9)
C18	0.0379 (10)	0.0417 (10)	0.0360 (10)	−0.0021 (8)	−0.0071 (8)	0.0024 (8)
C19	0.0438 (11)	0.0444 (11)	0.0482 (11)	0.0102 (9)	−0.0038 (9)	−0.0114 (9)
C20	0.0457 (11)	0.0451 (11)	0.0492 (11)	0.0061 (9)	−0.0118 (9)	−0.0176 (9)
C21	0.0392 (11)	0.0701 (15)	0.0692 (15)	0.0060 (11)	−0.0010 (10)	−0.0035 (12)
C22	0.0547 (13)	0.0710 (15)	0.0501 (13)	0.0037 (11)	0.0039 (10)	−0.0247 (11)
C23	0.0818 (16)	0.0473 (13)	0.0608 (14)	0.0158 (12)	−0.0029 (12)	0.0122 (11)
C24	0.0500 (12)	0.0444 (12)	0.0675 (14)	−0.0118 (9)	0.0000 (11)	0.0069 (10)
N1	0.0433 (9)	0.0453 (9)	0.0387 (9)	0.0044 (7)	−0.0038 (7)	−0.0107 (7)
N2	0.0369 (8)	0.0362 (8)	0.0411 (9)	−0.0048 (7)	0.0012 (7)	0.0017 (7)
N3	0.0475 (9)	0.0348 (8)	0.0427 (9)	0.0038 (7)	−0.0018 (7)	0.0043 (7)
O1	0.0501 (8)	0.0470 (8)	0.0548 (8)	0.0192 (6)	−0.0080 (7)	−0.0098 (7)
O2	0.0473 (7)	0.0394 (7)	0.0498 (8)	0.0078 (6)	0.0059 (6)	−0.0087 (6)
O3	0.0606 (9)	0.0849 (12)	0.0423 (8)	−0.0018 (8)	0.0053 (7)	0.0165 (8)
O4	0.0391 (8)	0.0582 (9)	0.0615 (9)	0.0001 (6)	−0.0094 (7)	−0.0099 (7)

### Geometric parameters (Å, °)

**Table:**

C1—N2	1.452 (2)	C13—N3	1.377 (2)
C1—C11	1.511 (2)	C14—O2	1.202 (2)
C1—C5	1.555 (2)	C14—N3	1.379 (2)
C1—C2	1.577 (2)	C15—C20	1.382 (2)
C2—C14	1.528 (2)	C15—C16	1.387 (2)
C2—C12	1.541 (2)	C16—C17	1.377 (3)
C2—C3	1.564 (2)	C16—H16	0.93
C3—C15	1.509 (2)	C17—C18	1.380 (3)
C3—C4	1.524 (3)	C17—H17	0.93
C3—H3	0.98	C18—O4	1.363 (2)
C4—N2	1.458 (2)	C18—C19	1.371 (3)
C4—H4A	0.97	C19—C20	1.386 (3)
C4—H4B	0.97	C19—H19	0.93
C5—O1	1.213 (2)	C20—H20	0.93
C5—N1	1.357 (2)	C21—O4	1.417 (2)
C6—C7	1.379 (3)	C21—H21A	0.96
C6—C11	1.383 (3)	C21—H21B	0.96
C6—N1	1.402 (2)	C21—H21C	0.96
C7—C8	1.384 (3)	C22—N1	1.453 (2)
C7—H7	0.93	C22—H22A	0.96
C8—C9	1.374 (3)	C22—H22B	0.96
C8—H8	0.93	C22—H22C	0.96
C9—C10	1.382 (3)	C23—N3	1.449 (2)
C9—H9	0.93	C23—H23A	0.96
C10—C11	1.377 (2)	C23—H23B	0.96
C10—H10	0.93	C23—H23C	0.96
C12—C13	1.495 (3)	C24—N2	1.457 (2)
C12—H12A	0.97	C24—H24A	0.96
C12—H12B	0.97	C24—H24B	0.96
C13—O3	1.206 (2)	C24—H24C	0.96
			
N2—C1—C11	114.62 (14)	O2—C14—C2	127.58 (17)
N2—C1—C5	113.10 (14)	N3—C14—C2	108.69 (15)
C11—C1—C5	100.82 (13)	C20—C15—C16	117.38 (16)
N2—C1—C2	102.35 (13)	C20—C15—C3	123.29 (16)
C11—C1—C2	116.31 (14)	C16—C15—C3	119.33 (16)
C5—C1—C2	110.02 (13)	C17—C16—C15	121.32 (17)
C14—C2—C12	101.07 (14)	C17—C16—H16	119.3
C14—C2—C3	109.14 (14)	C15—C16—H16	119.3
C12—C2—C3	120.23 (14)	C16—C17—C18	120.32 (17)
C14—C2—C1	108.66 (13)	C16—C17—H17	119.8
C12—C2—C1	113.20 (13)	C18—C17—H17	119.8
C3—C2—C1	104.19 (13)	O4—C18—C19	124.61 (17)
C15—C3—C4	115.57 (15)	O4—C18—C17	115.98 (16)
C15—C3—C2	116.42 (14)	C19—C18—C17	119.41 (17)
C4—C3—C2	104.44 (13)	C18—C19—C20	119.84 (18)
C15—C3—H3	106.6	C18—C19—H19	120.1
C4—C3—H3	106.6	C20—C19—H19	120.1
C2—C3—H3	106.6	C15—C20—C19	121.69 (17)
N2—C4—C3	103.73 (14)	C15—C20—H20	119.2
N2—C4—H4A	111.0	C19—C20—H20	119.2
C3—C4—H4A	111.0	O4—C21—H21A	109.5
N2—C4—H4B	111.0	O4—C21—H21B	109.5
C3—C4—H4B	111.0	H21A—C21—H21B	109.5
H4A—C4—H4B	109.0	O4—C21—H21C	109.5
O1—C5—N1	124.84 (17)	H21A—C21—H21C	109.5
O1—C5—C1	126.55 (16)	H21B—C21—H21C	109.5
N1—C5—C1	108.61 (15)	N1—C22—H22A	109.5
C7—C6—C11	122.24 (18)	N1—C22—H22B	109.5
C7—C6—N1	127.72 (18)	H22A—C22—H22B	109.5
C11—C6—N1	110.04 (15)	N1—C22—H22C	109.5
C6—C7—C8	117.1 (2)	H22A—C22—H22C	109.5
C6—C7—H7	121.5	H22B—C22—H22C	109.5
C8—C7—H7	121.5	N3—C23—H23A	109.5
C9—C8—C7	121.5 (2)	N3—C23—H23B	109.5
C9—C8—H8	119.3	H23A—C23—H23B	109.5
C7—C8—H8	119.3	N3—C23—H23C	109.5
C8—C9—C10	120.6 (2)	H23A—C23—H23C	109.5
C8—C9—H9	119.7	H23B—C23—H23C	109.5
C10—C9—H9	119.7	N2—C24—H24A	109.5
C11—C10—C9	118.96 (19)	N2—C24—H24B	109.5
C11—C10—H10	120.5	H24A—C24—H24B	109.5
C9—C10—H10	120.5	N2—C24—H24C	109.5
C10—C11—C6	119.59 (17)	H24A—C24—H24C	109.5
C10—C11—C1	131.08 (17)	H24B—C24—H24C	109.5
C6—C11—C1	109.33 (15)	C5—N1—C6	111.01 (15)
C13—C12—C2	104.72 (14)	C5—N1—C22	123.85 (17)
C13—C12—H12A	110.8	C6—N1—C22	125.11 (16)
C2—C12—H12A	110.8	C1—N2—C24	115.17 (15)
C13—C12—H12B	110.8	C1—N2—C4	106.52 (13)
C2—C12—H12B	110.8	C24—N2—C4	113.80 (15)
H12A—C12—H12B	108.9	C13—N3—C14	112.41 (15)
O3—C13—N3	124.28 (19)	C13—N3—C23	123.79 (17)
O3—C13—C12	128.21 (18)	C14—N3—C23	123.66 (16)
N3—C13—C12	107.50 (16)	C18—O4—C21	118.25 (16)
O2—C14—N3	123.71 (17)		
			
N2—C1—C2—C14	93.48 (15)	C3—C2—C14—O2	35.9 (2)
C11—C1—C2—C14	−32.26 (19)	C1—C2—C14—O2	−77.1 (2)
C5—C1—C2—C14	−146.03 (14)	C12—C2—C14—N3	−17.91 (17)
N2—C1—C2—C12	−155.10 (13)	C3—C2—C14—N3	−145.57 (14)
C11—C1—C2—C12	79.16 (18)	C1—C2—C14—N3	101.41 (15)
C5—C1—C2—C12	−34.61 (18)	C4—C3—C15—C20	−52.9 (2)
N2—C1—C2—C3	−22.77 (15)	C2—C3—C15—C20	70.3 (2)
C11—C1—C2—C3	−148.51 (14)	C4—C3—C15—C16	127.21 (18)
C5—C1—C2—C3	97.71 (15)	C2—C3—C15—C16	−109.68 (18)
C14—C2—C3—C15	113.00 (16)	C20—C15—C16—C17	−1.2 (3)
C12—C2—C3—C15	−2.9 (2)	C3—C15—C16—C17	178.77 (17)
C1—C2—C3—C15	−131.08 (15)	C15—C16—C17—C18	−0.7 (3)
C14—C2—C3—C4	−118.28 (15)	C16—C17—C18—O4	−178.00 (17)
C12—C2—C3—C4	125.78 (16)	C16—C17—C18—C19	2.2 (3)
C1—C2—C3—C4	−2.36 (17)	O4—C18—C19—C20	178.42 (18)
C15—C3—C4—N2	155.96 (14)	C17—C18—C19—C20	−1.8 (3)
C2—C3—C4—N2	26.73 (18)	C16—C15—C20—C19	1.6 (3)
N2—C1—C5—O1	52.8 (2)	C3—C15—C20—C19	−178.35 (18)
C11—C1—C5—O1	175.67 (18)	C18—C19—C20—C15	−0.1 (3)
C2—C1—C5—O1	−61.0 (2)	O1—C5—N1—C6	−177.45 (18)
N2—C1—C5—N1	−126.61 (16)	C1—C5—N1—C6	2.0 (2)
C11—C1—C5—N1	−3.74 (18)	O1—C5—N1—C22	0.8 (3)
C2—C1—C5—N1	119.62 (15)	C1—C5—N1—C22	−179.82 (17)
C11—C6—C7—C8	0.0 (3)	C7—C6—N1—C5	−179.8 (2)
N1—C6—C7—C8	−179.1 (2)	C11—C6—N1—C5	0.9 (2)
C6—C7—C8—C9	2.0 (4)	C7—C6—N1—C22	2.0 (3)
C7—C8—C9—C10	−1.5 (4)	C11—C6—N1—C22	−177.27 (18)
C8—C9—C10—C11	−1.1 (3)	C11—C1—N2—C24	−64.5 (2)
C9—C10—C11—C6	3.1 (3)	C5—C1—N2—C24	50.3 (2)
C9—C10—C11—C1	−176.66 (19)	C2—C1—N2—C24	168.64 (14)
C7—C6—C11—C10	−2.6 (3)	C11—C1—N2—C4	168.29 (15)
N1—C6—C11—C10	176.70 (16)	C5—C1—N2—C4	−76.88 (17)
C7—C6—C11—C1	177.21 (18)	C2—C1—N2—C4	41.45 (16)
N1—C6—C11—C1	−3.5 (2)	C3—C4—N2—C1	−43.87 (18)
N2—C1—C11—C10	−54.1 (3)	C3—C4—N2—C24	−171.88 (15)
C5—C1—C11—C10	−175.93 (19)	O3—C13—N3—C14	−170.56 (18)
C2—C1—C11—C10	65.2 (2)	C12—C13—N3—C14	10.2 (2)
N2—C1—C11—C6	126.08 (16)	O3—C13—N3—C23	5.3 (3)
C5—C1—C11—C6	4.28 (18)	C12—C13—N3—C23	−174.00 (18)
C2—C1—C11—C6	−114.63 (16)	O2—C14—N3—C13	−175.89 (17)
C14—C2—C12—C13	22.91 (17)	C2—C14—N3—C13	5.5 (2)
C3—C2—C12—C13	142.96 (15)	O2—C14—N3—C23	8.3 (3)
C1—C2—C12—C13	−93.10 (16)	C2—C14—N3—C23	−170.30 (17)
C2—C12—C13—O3	159.54 (19)	C19—C18—O4—C21	−5.5 (3)
C2—C12—C13—N3	−21.23 (19)	C17—C18—O4—C21	174.65 (18)
C12—C2—C14—O2	163.58 (17)		

### Hydrogen-bond geometry (Å, °)

**Table:**

*D*—H···*A*	*D*—H	H···*A*	*D*···*A*	*D*—H···*A*
C10—H10···O2	0.93	2.52	3.122 (2)	123
C12—H12A···O1	0.97	2.58	3.222 (2)	124
C20—H20···O1	0.93	2.56	3.400 (2)	150
C12—H12B···O1^i^	0.97	2.56	3.264 (2)	129
C16—H16···O4^ii^	0.93	2.50	3.394 (2)	161
C19—H19···O2^iii^	0.93	2.54	3.440 (2)	162
C21—H21C···Cg1^iv^	0.96	2.93	3.592 (2)	127

Symmetry codes: (i) −*x*−1/2, *y*−1/2, *z*; (ii) *x*+1/2, −*y*+1/2, −*z*; (iii) −*x*−1/2, *y*+1/2, *z*; (iv) −*x*+1, −*y*+1, −*z*.
